# Cervical cancer screening uptake and challenges in Malawi from 2011 to 2015: retrospective cohort study

**DOI:** 10.1186/s12889-016-3530-y

**Published:** 2016-08-17

**Authors:** Kelias Phiri Msyamboza, Twambilire Phiri, Wesley Sichali, Willy Kwenda, Fanny Kachale

**Affiliations:** 1 World Health Organization, Malawi Country Office, Lilongwe, Malawi; 2 Ministry of Health, Reproductive Health Directorate, Lilongwe, Malawi; 3 Ministry of Health, Mzimba North District Hospital Office, Mzuzu, Malawi; 4 Ministry of Health, Balaka District Hospital Office, Balaka, Malawi; 5 WHO Malawi, ADL House, City Centre, P.O. Box 30390, Lilongwe, Malawi

**Keywords:** Cervical cancer, Visual inspection with acetic acid, Cryotherapy, Sub-Saharan Africa, Malawi

## Abstract

**Background:**

Malawi has the highest cervical cancer incidence and mortality in the world with age-standardized rate (ASR) of 75.9 and 49.8 per 100,000 population respectively. In response, Ministry of Health established a cervical cancer screening programme using visual inspection with acetic acid (VIA) and treatment of precancerous lesions with cryotherapy. This paper highlights the roll out, integration with family planning services and HIV ART Programme, uptake and challenges of VIA and Cryotherapy programme.

**Methods:**

We analyzed program data, supportive supervision, quarterly and annual reports from the National Cervical Cancer Control Program. We evaluated the uptake and challenges of screening services by age, HIV serostatus and trends over a five year period (2011–2015).

**Results:**

Between 2011 and 2015, number of cervical cancer screening sites, number of women screened and coverage per annum increased from 75 to 130, 15,331 to 49,301 and 9.3 % to 26.5 % respectively. In this five year period, a total of 145,015 women were screened. Of these, 7,349 (5.1 %) and 6,289 (4.3 %) were VIA positive and suspect cancer respectively. Overall 13,638 (9.4 %) were detected to be VIA positive or had suspect cancer. Of the 48,588 women with known age screened in 2015; 13,642 (28.1 %), 27,275 (56.1 %) and 7,671 (15.8 %) were aged 29 or less, 30–45, 46 years or more. Among 39,101 women with data on HIV serostatus; 21,546 (55.1 %) were HIV negative, 6,209 (15.9 %) were HIV positive and 11, 346 (29.0 %) status was unknown. VIA positivity rate and prevalence of suspect cancer were significantly higher in HIV positive than HIV negative women (8.8 % vs 5.0 %, 6.4 % vs 3.0 %); in women aged 30–45 years than women aged 29 years or less (5.6 % vs 2.3 %, 2.6 % vs 1.2 %) respectively, all *p* <0.05). The main challenge of the programme was failure to treat VIA positive women eligible for cryotherapy. Over the five year period, the programme only treated 1,001 (43.3 %) out of 2,311 eligible women and only 266 (31.8 %) of the 836 women with large lesion or suspect cancer who were referred, received the health care at the referral centre. The reasons for failure to provide cryotherapy treatment were stock out of gas, faulty/broken cryotherapy machine (usually connectors or probes) or no cryotherapy machine at all in the whole district. For women with large lesion or suspect cancer; lack of loop electrosurgical excision procedure (LEEP) machine or inadequate gynaecologists at the referral centre, were the major reasons. Cancer radiotherapy services were not available in Malawi.

**Conclusions:**

This study provided data on VIA positivity rate, prevalence of suspect cancer, failure rate of cryotherapy and challenges in the provision of cryotherapy and LEEP treatment in Malawi. These data could be used as baseline for monitoring and evaluation of Human Papillomavirus (HPV) vaccination programme which the country introduced in 2013, the linkage of cervical cancer screening and women on HIV ART and the long term effect of ART, voluntary male medical circumcision on the prevalence and incidence of cervical cancer.

## Background

Malawi has the highest cervical cancer incidence and mortality in the world with age-standardized rate (ASR) of 75.9 and 49.8 per 100,000 population respectively [1]. Cervical Cancer accounts for 45.4 % of all cancers in women and the trend is increasing [2]. Every year, over 2,300 women develop cervical cancer and over 1,600 die from the disease [3]. The median survival time from the time of diagnosis is 10 months and 5-year survival rate is 2.9 % [4]. High prevalence of HIV (10.6 %), human papilloma virus (33.6 %), inadequate screening and treatment services for precancerous lesions, late diagnosis, limited access to timely, standard treatment of cancer and palliative care are the main risk factors for high cervical cancer incidence and mortality [5–8].

In response to high burden of cervical cancer, the Malawi Ministry of Health, through Sexual and Reproductive Health Directorate is implementing human papillomavirus (HPV) vaccination pilot project and screen-and-treat programme using visual inspection with acetic acid (VIA) and cryotherapy. The cervical cancer screening programme started in 2004 as a pilot project in Mulanje district. The programme is integrated with family planning services and targets women aged 30–45 years and women on HIV ART. HIV negative women are screened once every five years while HIV positive are screened once every 2–3 years. By 2011 the programme was scaled up to all central and district hospitals, most mission hospitals and some health centres and private hospitals. There were over 130 VIA, 32 cryotherapy, 11 cold coagulation and 3 loop electrosurgical excision procedure (LEEP) sites in public health facilities across the country [9]. Malawi is one of the few countries in east and southern Africa with high burden of cervical cancer that have rolled out screen-and- treat programme nationally. However data on successes and challenges of the Malawi national programme have not been documented before. This paper therefore aims to contribute in filling the information gap on the uptake, outcome by age and HIV sero-status, overall cure rate and challenges of VIA and Cryotherapy programme over a five year period (2011–2015).

## Methods

### Study type

This was a retrospective cohort study of women attending cervical cancer screening sites for initial visit, sub-sequent visit and follow up. Analysis of the national cervical cancer control programme database and reports of national, zonal, and district supportive supervision and annual programme review meetings for the period 2011–2015 was conducted.

### Data management

Data was entered in Microsoft excel® and exported to SPSS version 20 for analysis. Some of the variables analyzed were: trend in the number of women screened over 5-year period, VIA positivity rate and prevalence of suspect cancer among women screened by age and HIV status, proportion of women who actually received cryotherapy among those who were eligible, loss to follow up among women eligible for cryotherapy and overall cure rate at 1- year for cryotherapy. According to Malawi National Statistics Office (NSO) population figures, women aged 30–45 years (the target group) constitute 6.74 % of the total population [5]. The target for the national cervical cancer programme is to screen at least 80 % of the target population over five years. Therefore the annual target was calculated as total population multiplied by 0.0674 multiplied by 0.8 divided by 5. The annual coverage (%) was therefore calculated by dividing the total number of women screened per year by the annual target multiplied by 100. Confidence intervals (CI) for proportion were calculated using the formula *p* = p ± C√p(1-p)/n where p is the given proportion whose CI needs to be calculated, C is the coefficient, at 95 % CI, C = 1.96, *n* = number of participants. The results were statistically significant if Chi-square *p* < 0.05 or there was no overlap between two CIs of comparing groups; HIV negative vs HIV positive, age 30–45 years vs age ≤29 years, or ≥46 years. All analyses were made at 95 % confidence level.

## Results

### Number of cervical cancer screening and treatments sites and providers

As of 31 December 2015, there were a total of 130 (an increase from 75 in 2011) VIA and 1 Pap smear (Malamulo Makwasa Hospital) public sites that were conducting cervical cancer screening countrywide. The screening services were available (at least at district hospital) in all the districts except one (Likoma Island). There were 32 established cryotherapy sites. Of these 10 were not functional because the machine was faulty, mainly the connectors or the probes were broken (7 sites) or lack of gas (3 sites). Four cryotherapy sites which were not functional (Rumphi, Nkhatabay, Mulanje and Phalombe) were in the districts where there was no any other site to treat VIA positive eligible cases and women were referred to another district. One district (Ntchisi) never had cryotherapy although it was providing screening services. In addition to 22 functional cryotherapy sites, they were 11 cold (thermo) coagulation sites which were introduced recently (2013–2015) as a new treatment method for pre-cancerous cervical cancer lesions. There were a total of 395 VIA and cryotherapy providers who were trained during the period 2011–2015. Of these, 276 (69.9 %) were active and providing the services. On average therefore, there were 2 (276/130) active providers per VIA site.

### Number of women screened and their outcomes

In 2015, at total of 49,301 women were screened for cervical cancer countrywide. Of these, 44,951 (91.2 %) were initial, 1,487 (3.0 %) were one year follow up after cryotherapy and 2,857 (5.8 %) was sub-subsequent visit at five years after VIA negative result at the initial screening. In total there were 2,311 (4.7 %), 2,082 (4.6 %), 105 (7.1 %) and 124 (4.3 %) VIA positive women among all women screened, initial, one year follow up and sub-sequent respectively. Of the 2,311 VIA positive women who were eligible for cryotherapy, only 1,001 (43.3 %) received cryotherapy. Among 1,001 cryotherapy conducted, 562 (56.1 %) were done immediately (screen-and-treat) 439 (43.9 %) were post-postponed and done weeks or months later. Over 500 cryotherapy clients who were post-postponed were never done. Lack of cryotherapy machine, gas or faulty machine were the common reasons for failure to treat VIA positive eligible women. Of the 836 women who were referred to another facility for cryotherapy or large lesion, only 266 (31.8 %) reported back to the facilities that referred them after receiving treatment at the referral hospital. The prevalence of suspect cancer among initial was 1,665 (3.7 %). There were no suspect cancer cases among one year follow and sub-sequent visits.

### Number of women screened by age and HIV serostatus

Of the 48,588 women with known age, 13,642 (28.1 %), 27,275 (56.1 %) and 7,671 (15.8 %) were aged 29 or less, 30–45, 46 years or more respectively. VIA positivity rate was: 2.3 %, 5.6 % and 5.2 % while prevalence of suspect cancer was 1.2 %, 2.6 % and 11.9 % respectively. Statistically, VIA positivity rate was significantly lower in women aged 29 years or less and prevalence of suspect cancer was higher in women aged 46 years or more compared to women aged 30–45 years, all *p* < 0.05.

Among 39,101 women with data on HIV sero-status, 21,546 (55.1 %) were HIV negative, 6,209 (15.9 %) were positive and 11,346 (29.0 %) the status was unknown. Prevalence of VIA positivity and suspect cancer was higher in HIV positive than HIV negative women, 8.8 % vs 5.0 %, 6.4 % vs 3.0 % respectively, all *p* < 0.05 (Table 1).

**Table 1:** Characteristics and outcome of women screened in 2015 by age and HIV status

	Total		VIA positive			Suspect cancer			VIA positive or suspect cancer		
	n	%	n	%	95 % CI	n	%	95 % CI	n	%	95 % CI
Age (years):											
≤ 29	13,642	28.1	307	2.3*	1.4-3.2	163	1.2*	0.3-2.1	470	3.4*	2.6-4.2
30-45	27,275	56.1	1,540	5.6 (ref)	5.0-6.2	710	2.6 (ref)	2.0-3.2	2,250	8.2 (ref)	7.6-8.8
≥ 46	7,671	15.8	399	5.2	4.1-6.3	910	11.9*	10.8-13.0	1,309	17.1*	16.1-18.1
All with known age	48,588	100.0	2,246	4.6	4.2-5.0	1,783	3.7	3.3-4.1	4,029	8.3	7.9-8.7
HIV status:											
Negative	21,546	55.1	1,088	5.0 (ref)	4.3-5.7	638	3.0 (ref)	2.3-3.7	1,726	8.0 (ref)	7.3-8.7
Positive	6,209	15.9	546	8.8*	7.6-10.0	399	6.4*	5.2-7.6	945	15.2*	14.0-16.4
Unknown	11,346	29.0	558	4.9	4.0-5.8	520	4.6*	3.7-5.5	1,078	9.5*	8.6-10.4
All with HIV status data	39,101	100.0	2,192	5.6	5.1-6.1	1,557	4.0	3.5-4.5	3,749	9.6	9.1-10.1

CI = confidence interval, *n* = number of women in the group, VIA = visual inspection with acetic acid, % = percentage, ref = reference, * = statistically significant, *p* < 0.05

### Trends in cervical screening 2011–2015

Number women screened for cervical cancer increased by year from 15,331 in 2011 to 49,301 in 2015. Coverage of the eligible population (women aged 30–45 years, 6.74 % of total population, programme target 80 % to be screened in 5 years) increased from 9.3 % in 2011 to 26.5 % by the end of 2015. A total of 145,015 women were screened of whom 5.1 % and 4.3 % were VIA positive, had suspect cancer respectively. Only 40.4 % of 7,349 VIA positive women who were eligible for cryotherapy were actually treated. Positivity rate of VIA and prevalence of suspect cancer remained around 5 % and 4 % respectively (Table 2).

**Table 2:** Trends in cervical screening in Malawi 2011-2015

Year	Annual target	Number of women screened		Number of women with VIA positive		Number of women with VIA positive treated with cryo or cold coagulation		Number of women with suspect cancer	
		n	% coverage^a^	n	%	n	%	n	%
2011	164,145	15,331	9.3	894	5.8	388	43.4	798	5.2
2012	169,350	22,400	13.2	1,069	4.8	400	37.4	1,098	4.9
2013	174,735	20,490	11.7	1,447	7.1	528	36.5	1,294	6.3
2014	180,306	37,493	20.8	1,628	4.3	655	40.2	1,434	3.8
2015	186,069	49,301	26.5	2,311	4.7	1,001	43.3	1,665	3.4
Total		145,015	-	7,349	5.1	2,972	40.4	6,289	4.3

Cryo = Cryotherapy, *n* = number of women in the group, VIA = visual inspection with acetic acid, % = percentage

^a^Annual coverage (%) = Number of women screened in a year/Annual target ×100

Annual target = Total population × 0.0674 × 0.8/ 5

## Discussion

This was the first study in Malawi and one of the few studies from countries in east and southern Africa where the burden of cervical cancer is high, that has provided comprehensive data on the roll out, successes and challenges of the cervical cancer screening and treatment of pre-cancerous lesions. The study has documented that Malawi successfully scaled up cervical cancer screening services using visual inspection with acetic acid (VIA). In the period 2011–2015, the number of cervical cancer screening sites, number of women screened and coverage per annum increased from 75–130, 15,331–49,301 and 9.3–26.5 % respectively. A total of 145,015 women were screened for at least once. Cervical cancer screening, even when done once, reduces the burden of cervical cancer mortality. It is estimated that in countries with high burden of HIV and cervical cancer, one-time cervical cancer screening prevents one death for every 202 women screened [10, 11]. Based on this estimate, at least 718 deaths due to cervical cancer were therefore averted.

This study has also demonstrated that the VIA positivity rate and cryotherapy failure rate were similar to those reported in Tanzania, Mozambique and systematic reviews [12–16] but lower to what was reported in Zambia and Kenya where VIA positivity rates were 17–20 % [17–19]. Majority of women in Zambian and Kenyan studies were HIV positive and therefore this could explain the high positivity rate.

It is well documented that HIV-positive women, compared to HIV negative women, women aged 30–45 years compared to women aged 29 years have a higher prevalence of cervical precancerous lesions as well as a faster progression of these lesions to invasive cancer [20]. This study was in agreement with these findings. VIA positivity rate and prevalence of suspect cancer were significantly higher in HIV positive than HIV negative women (8.8 % vs 5.0 %, 6.4 % vs 3.0 %) in women aged 30–45 years than women aged 29 years or less (5.6 % vs 2.3 %, 2.6 % vs 1.2 %) respectively, all *p* <0.05).

However, despite the aforementioned successes of the Malawi National Cervical Cancer Screening Programme, the programme faced a number of challenges. The main challenge was failure to treat VIA positive women eligible for cryotherapy. Over the five year period, the programme only treated 1,001 (43.3 %) out of 2,311 eligible women. The problem was even worse for women with large lesions or suspect cancer who were referred to central hospitals for further assessment and management. Of the 836 women who were referred, only 266 (31.8 %) received the service at the referral centre. The main reasons for failure to provide cryotherapy treatment were stock out of gas, faulty/broken cryotherapy machine (usually connectors or probe) or no cryotherapy machine at all in the whole district. For women with large lesions or suspect cancer; lack of LEEP machine or inadequate gynaecologists at the central hospitals, were the major reasons. Cancer radiotherapy services were not available in Malawi. Similar health system, equipment and referral challenges were documented in Mozambique and Kenya [13, 19]. This study therefore contributed in documenting evidence on the challenges faced in the provision of cryotherapy treatment although it is considered to be affordable, effective and recommended for resource- poor countries by WHO. In Malawi, cryotherapy had a high running cost (gas, connectors and probes) and districts were failing to sustain the service. The three year experience from the 11 sites that were providing cold (thermo) coagulation showed that cold coagulation had no problems, low running cost, easy to use by nurses and all eligible women received the treatment [21]. All public hospitals have relatively stable electricity and therefore cold coagulation may be the way forward to high running cost of cryotherapy treatment in a resource- poor setting [22].

The other challenge that the programme experienced was the high (32 %) loss to follow for a repeat cryotherapy at one year follow up among women who tested VIA positive and were done cryotherapy. With cryotherapy failure rate of 7 %, it would be important to provide evidence- informed counseling and strategies to reduce the loss to follow up. High loss to follow up was also reported in Kenya [19]. High staff turnover, though not peculiar to this programme but a general problem in ministry of health, was another challenge where up to 30 % of health workers that were trained were no longer providing the cervical cancer screening services.

Lastly, although coverage of women screened for cervical cancer per annum increased from 9 % in 2011 to 27 % in 2015, it was still far below the programme target of 80 %. Concerted efforts by government, development partners and non-governmental organizations to address the above named challenges may improve the coverage.

### Limitations of the study

This study made use of the available programme health facility data and reports on cervical cancer screening and treatment. The use of health facility data has its own limitations, such as incompleteness and bias in the sense that the information is obtained only from people who came to health facilities or clinics. These limitations were not peculiar to this study but would affect any other study that uses facility data. Nonetheless, this comprehensive documentation has contributed in providing information on the successes and challenges of VIA and cryotherapy programme in a resource-poor setting.

## Conclusions

This study has provided data on VIA positivity rate, prevalence of suspect cancer, failure rate of cryotherapy and challenges in the provision of cryotherapy and LEEP treatment in Malawi. These data could be used as baseline for monitoring and evaluation of Human Papillomavirus (HPV) vaccination programme which was introduced in 2013, the linkage of cervical cancer screening and women on HIV ART and the long term effect of ART, voluntary male medical circumcision or a combination of these programmes on the prevalence and incidence of cervical cancer. Concerted efforts by government, development partners and non-governmental organizations to address the challenges faced by the programme may improve the coverage. Because of high running cost of cryotherapy, cold coagulation could be considered as an alternative treatment method in Malawi, a poor country in central-southern Africa.
